# Reduced Efficacy of Insecticide-treated Nets and Indoor Residual Spraying for Malaria Control in Pyrethroid Resistance Area, Benin

**DOI:** 10.3201/eid1302.060631

**Published:** 2007-02

**Authors:** Raphael N’Guessan, Vincent Corbel, Martin Akogbéto, Mark Rowland

**Affiliations:** * London School of Hygiene and Tropical Medicine, Cotonou, Benin, West Africa; † Institut de Recherche pour le Developpement, Montpellier, France; ‡ University of Abomey-Calavi, Cotonou, Benin; § Centre de Recherche Entomologique, Cotonou, Benin; ¶ London School of Hygiene and Tropical Medicine, London, United Kingdom

**Keywords:** *Anopheles gambiae*, pyrethroid resistance, insecticide treated net, indoor residual spraying, research

## Abstract

The pyrethroid knockdown resistance gene (*kdr*) has become widespread in *Anopheles gambiae* in West Africa. A trial to test the continuing efficacy of insecticide-treated nets (ITN) and indoor residual spraying (IRS) was undertaken in experimental huts at 2 sites in Benin, 1 where *kdr* is present at high frequency (Ladji), the other where *An. gambiae* is susceptible (Malanville). Holes were made in the nets to mimic worn nets. At Malanville, 96% of susceptible *An. gambiae* were inhibited from blood-feeding, whereas at Ladji feeding was uninhibited by ITNs. The mortality rate of *An. gambiae* in ITN huts was 98% in Malanville but only 30% at Ladji. The efficacy of IRS was equally compromised. Mosquitoes at Ladji had higher oxidase and esterase activity than a laboratory-susceptible strain, but this fact did not seem to contribute to resistance. Pyrethroid resistance in *An. gambiae* appears to threaten the future of ITN and IRS in Benin.

## Abstract 2

These tools may no longer be effective for malaria control in parts of Benin.

During the last decade, pyrethroid-treated mosquito nets have become the main method of malaria prevention in many malaria-endemic African countries (*1*,*2*). In a few notable exceptions, usually those with a more developed health infrastructure, such as South Africa, a longstanding practice of applying indoor residual spraying (IRS) has been successful (*3*). The 2 approaches to malaria prevention, insecticide-treated nets (ITNs) and spraying (IRS), are not mutually exclusive, and in malaria-endemic areas where ITN coverage is still limited, the feasibility of introducing IRS to reduce transmission is being considered, for example, by the President’s Initiative Fund (*4*). Trials of IRS and ITNs have shown that in areas with pyrethroid-susceptible *Anopheles gambiae* the effectiveness of the 2 methods in controlling malaria does not differ (*5*). This comparability may not hold true for areas with pyrethroid-resistant populations. In southern Africa, for example, IRS with pyrethroid failed to control pyrethroid-resistant *An. funestus* and necessitated a switch to an alternative class of insecticide to which there was no resistance (*6*). During the last decade, pyrethroid resistance caused by the *kdr* mechanism has become widespread in *An. gambiae* in West Africa and is common in some areas (*7*). Whether *kdr* undermines the effectiveness of ITN in areas of high prevalence is unclear. An early experimental hut trial of ITNs in Côte d’Ivoire demonstrated a survival advantage of homozygotes for *kdr* resistance (*8*), whereas subsequent hut trials in adjacent resistant and susceptible populations showed no apparent difference in the effectiveness of ITNs between the 2 localities (*9*). Village randomized trials in Côte d’Ivoire showed that ITNs continued to prevent malaria despite a vector population that was *kdr* resistant (*10*). Whether *kdr* would undermine the effectiveness of IRS in the same way as resistance due to oxidases did against *An. funestus* in southern Africa (*6*) is unknown. To assess the practicability of applying IRS with pyrethroid in West Africa, we need to examine the effectiveness of this approach against a *kdr-*resistant population of *An. gambiae* is important. To get a clearer understanding of the influence of *kdr* resistance on the effectiveness of ITN, further experimental hut trials of ITNs against *kdr*-resistant populations need to be conducted. We describe 2 experimental hut trials in Benin. One compares the impact of IRS and ITN against a *kd-* resistant population in the southern part of the country; the other compares IRS and ITNs against a pyrethroid-resistant population several hundred kilometers to the north.

## Material and Methods

### Study Sites

Ladji is a large village on the outskirts of Cotonou, the capital of Benin. The village floods during the rainy season. *An. gambiae* comprises the Mopti (M) cytotype and shows resistance to pyrethroids and DDT; *kdr* is present at high frequency (*11*). The nuisance mosquito *Culex quinquefasciatus* is also present and shows resistance to pyrethroids. Five experimental huts belonging to the Centre de Recherche Entomologique de Cotonou (CREC) are situated in the village.

Malanville is in northern Benin, 800 km from Cotonou, in an irrigated rice-growing valley. The local *An. gambiae* comprises the M cytotype, but the *kdr* gene is almost absent and mosquitoes are susceptible to lambdacyalothrin and deltamethrin. Six experimental huts are present at Malanville.

### Experimental Huts

The treated nets, residual spray treatments, and their respective untreated controls were evaluated in 4 experimental huts at each field site. Experimental huts are specially designed to test vector control product against freely entering mosquitoes under natural but controlled conditions. Huts were typical of the region. Each was made from concrete bricks, with a corrugated iron roof and a ceiling of thick polyethylene sheeting lined with hessian sackcloth on the interior surface, and each was built on a concrete base surrounded by a water-filled moat to exclude ants (*12*). Mosquito access was through 4 window slits, constructed from pieces of plywood fixed at an angle to create a funnel with a 1-cm gap, present on 3 sides of the huts. Mosquitoes had to fly upward to enter through the gaps and downwards to exit; this precluded or limited exodus through the aperture and enabled us to account for most entering mosquitoes. A veranda trap projected from the back wall of each hut. Movement of mosquitoes between a room and the veranda was unimpeded.

### Mosquito Net Treatments

The nets were made of white, 100-denier polyester (SiamDutch Mosquito Netting Co., Bangkok, Thailand). Nets measured 2.0-m long, 1.6-m wide, and 1.8-m tall and had a surface area of 16.9 m^2^. To simulate badly torn nets, 80 holes, each measuring 2 × 2 cm, were cut in the sides and ends of each net.

Insecticides used were formulations of lambdacyhalothrin (Icon, Syngenta, Switzerland): lambdacyhalothrin 2.5% CS, a microencapsulated suspension designed for ITNs, and lambdacyhalothrin 10% WP, a wettable powder designed for IRS.

The lambdacyhalothin application rates of 18 mg/m^2^ for ITNs and 30 mg/m^2^ for IRS were within the ranges recommended by the manufacturer. Indoor residual treatments were applied with a hand-operated compression sprayer equipped with a flat fan nozzle. The cement walls and sackcloth ceilings were sprayed uniformly after masking the veranda and window slits with protective coverings. The control hut was sprayed with water only. The treated huts were left for 1 week before evaluations were started.

### Sleepers and Mosquito Collections

Preliminary experiments showed the huts to be evenly attractive to mosquitoes. The treatments were randomly allocated to the 4 experimental huts at each site. The main trials were conducted from April to June 2005 at the Ladji site and from September to November 2005 at the Malanville site. Eight adult men employed by CREC slept overnight in the huts and collected mosquitoes from the huts in the mornings. Informed consent to participate in the study was given beforehand, and chemoprophylaxis was provided during the trial. Ethical approval was granted by the London School of Hygiene and Tropical Medicine (LSHTM) and Benin national ethics committees.

The trial ran for 50 nights for 8 weeks at each site. The sleepers were rotated between huts to correct for possible variation in individual attractiveness. Each morning, mosquitoes were collected from the floors, walls, and ceilings of rooms, verandas, and nets with aspirators and torches. Mosquitoes were identified and scored as blood-fed or unfed and dead or live. Live mosquitoes were held in netted plastic cups and supplied with 10% honey solution for 24 h before delayed death was recorded. Male mosquitoes were not recorded.

The entomologic impact of each treatment on mosquitoes was expressed relative to the control in terms of the following: deterrence, the proportional reduction in the number of mosquitoes entering a treated hut relative to that entering the control hut; induced exophily, the proportion of mosquitoes collected from the veranda trap of the treatment hut relative to the proportion in the veranda of the control hut; blood-feeding inhibition, the reduction in blood-feeding rate relative to the control hut; and mortality, the proportions of mosquitoes found dead in the hut at the time of collection and after a 24-h holding period.

If a treatment deters a considerable number of mosquitoes from entering the hut, the values given by proportion blood-feeding or proportion killed in the treatment hut may underestimate the full personal protective effect and overestimate the full insecticidal efficacy of the treatment. The personal protective effect of a treatment is better described by the reduction in the number of blood-fed mosquitoes in the treatment hut relative to the number blood-fed in the control hut:

% personal protection = 100 (B_u_ – B_t_)/B_u_

where B_u =_ is the total number of blood-fed mosquitoes in the untreated control huts and B_t_ is the total number blood-fed mosquitoes in the huts with insecticide treatment.

The overall insecticidal effect of a treatment needs to take into account that a considerable number of mosquitoes might be deterred from entering the hut and hence not be killed by the treatment. A mass killing effect is desirable to reduce transmission. The overall insecticidal effect of a treatment relative to the number of mosquitoes that would ordinarily enter an untreated hut can be estimated by using the following formula and expressed as a percentage:

Overall insecticidal effect (%) = 100 (K_t_ – K_u_)/(T_u_ – K_u_)

where K_t_ is the number killed in the treated hut, K_u_ is the number dying in the untreated control hut, and T_u_ is the total number collected from the control hut.

### Residual Activity of Insecticide Treatments

To evaluate residual activity, World Health Organization (WHO) cone bioassays were undertaken monthly in the Ladji huts and bimonthly in the Malanville huts with a laboratory-susceptible strain of *An. gambiae* (Kisumu). *An. gambiae* females, 3–5 days old, were exposed within the cones to nets for 3 min or to sprayed walls and ceilings for 30 min. Approximately 50 mosquitoes in 5 replicates of 10 mosquitoes were tested on each substrate. Honey solution was provided during the 24-h holding period, and the temperature was kept at 25°C.

### Biochemical Assays

Biochemical tests on individual mosquitoes were conducted to determine the activity of mixed function oxidases and nonspecific esterases present in pyrethroid-resistant and -susceptible samples of *An. gambiae* from the Ladji and Malanville sites. Tests were conducted on 3-day-old adult females (initially collected as larvae) in microtiter plates (*13*). Susceptible (Kisumu) and pyrethroid-resistant (Vkper) *An. gambiae* served as controls. Genotyping of *An. gambiae* was carried out to assess *kdr* frequency at both field stations (*14*).

### Adult Bioassay Data

To determine whether a stronger pyrethroid resistance mechanism was present in the Ladji population than in the standard *kdr* strain Vkper, bioassays with 0.05% lambdacyalothrin-treated papers (18 mg/m^2^) were conducted in WHO resistance test kits by using a range of exposure times on batches of 25 unfed *An. gambiae* females 2–5 days of age. One hundred mosquitoes per exposure period were tested. Deaths were scored 24 h later. Log-time mortality curves were generated, and lethal time to kill 50% (LT_50_), estimated by using probit analysis.

### Data Analysis

Proportional data from the hut trial (exophily, blood-feeding, deaths) were analyzed by using logistic regression (STATA 6 software**,** Stata Corporation, College Station, TX, USA). Deterrence rates were analyzed by comparing the number of mosquitoes entering each hut by using the Wilcoxon rank sum test. Biochemical activity was analyzed with Kruskal–Wallis and Wilcoxon rank sum tests. The level of resistance to lambdacyalothrin in insecticide bioassays was analyzed by using probit analysis.

## Results

### Insecticide Residual Activity

Residual activity on ITN as measured by cone bioassay tests showed no decline during the 8 weeks of the trial. Activity of the IRS wettable powder formulation on sackcloth and cement showed a decline in performance by week 4. This trend continued until the end of the trial (Table 1).

**Table 1:** Residual activity of lambdacyalothrin (insecticide)-treated nets (ITNs) and indoor residual spraying over 3 mo in experimental huts, Malanville and Ladji field stations*

When and where substrate tested	ITNs at 18 mg/m^2^		Indoor residual spraying at 30 mg/m^2^			
	Sides + top of net		Ceiling		Walls	
	No. tested	% Corrected mortality	No. tested	% Corrected mortality	No. tested	% Corrected mortality
Wk 0						
Malanville	77	100	33	100	60	100
Ladji	51	100	30	93.3	54	100
Wk 2						
Ladji	52	100	22	100	41	100
Wk 4						
Ladji	54	100	21	52.4	47	42.5
Wk 6						
Ladji	57	100	25	80.0	45	31.1
Wk 8						
Malanville	52	100	29	41.4	54	2.6
Ladji	44	97.7	8	25.0	39	18.5

*As determined by using World Health Organization cone bioassays and susceptible *Anopheles gambiae* (Kisumu).

### Efficacy of Treatments in Huts

Over the 2-month trial, 1,395 *An. gambiae*, 3,070 *Cx. quinquefasciatus,* and small numbers of *Mansonia uniformis*, *An. pharoensis,* and *Aedes aegypti* were collected at Ladji. At Malanvile, 1,523 *An. gambiae*, 2,804 *Mansonia* sp., and smaller numbers of *An. funestus* and *Ae. aegypti* were collected. Only the malaria vector *An. gambiae* and the nuisance mosquito *Cx. quinquefasciatus* were analyzed further.

Fewer *An. gambiae* entered the ITN- and IRS-treated huts than the respective control huts. The treatment induced reduction in hut entry was more evident in the resistance area than in the susceptible area (Table 2). The proportion deterred at each site did not differ between ITN or ITS treatments.

**Table 2:** Experimental hut results of lambdacyhalothrin (insecticide)-treated nets (ITNs) and indoor residual spraying (IRS) against *Anopheles gambiae,* Ladji (pyrethroid resistance) and Malanville (pyrethroid susceptibility) field stations*

		Ladji (pyrethroid resistance)		Malanville (pyrethroid susceptibility)	
ITN		Untreated net	Lambdacyhalothrin 18 mg/m^2^	Untreated net	Lambdacyhalothrin 18 mg/m^2^
	Total collected	689^a^	386^b^	363^a^	267^b^
	Deterred, %	_	44.0	._	26.4
	Exiting, % (CI)	25.0† (21.7–28.2)	29.0† (24.5–33.5)	36.1 (31.1–41.0)	46.8‡ (40.8–52.8)
	Blood-fed, % (CI)	82.0† (79.1–84.9)	82.1† (78.3–85.9)	77.7† (73.4–81.9)	3.0‡ (0.9–5.0)
	Blood-feeding inhibition, %	_	0	_	96.1
	Personal protection, % (no. bloodfed)	– (572)	44.6 (317)	– (282)	97.2 (8)
	% Dead (CI)	13.6† (11.1–16.2)	29.8‡ 25.2–34.4)	3.6† (1.7–5.5)	98.5‡ (97.0–99.9)
	Insecticidal effect, % (no. dead)	– (94)	3.0 (115)	– (13)	68.9 (263)
IRS		Unsprayed hut	Lambdacyhalothrin 30 mg/m^2^	Unsprayed hut	Lambdacyhalothrin 30 mg/m^2^
	Total collected	203†	117‡	498†	395‡
	Deterred, %	_	42.4	_	20.7
	Exiting, % (CI)	45.8† (38.9–52.7)	58.1† (49.2–67.1)	54.4† (50.0–58.8)	63.3† (58.5–68.0)
	Blood-fed, % (CI)	87.7† (83.2–92.2)	73.5‡ (65.5–81.5)	93.8† (91.6–95.9)	69.6‡ (65.1–74.2)
	Blood-feeding inhibition, %	_	16.2	_	25.8
	Personal protection, % (no. bloodfed)	– (178)	51.7 (86)	– (467)	41.1 (275)
	Dead, % (CI)	12.3† (7.8–16.8)	30.8† (22.4–39.1)	1.4† (0.4–2.4)	72.1‡ (67.7–76.6)
	Insecticidal effect, % (no. dead)	– (25)	5.4 (36)	– (7)	55.8 (285)

*For each untreated–treated pair, values not sharing the same superscript are significantly different at the 5% level.. CI, 95% confidence interval.

The untreated net was little or no barrier to blood-feeding of *An. gambiae* at either field site owing to the large number of holes cut in each net. Treating the holed net with pyrethroid led to a 96% reduction in the number of mosquitoes blood-feeding at the susceptible site (Malanville) but to no reduction in blood-feeding at the resistant site (Ladji). Inhibition of blood-feeding by IRS at either the resistant or susceptible site was limited (Table 2).

Natural mortality of *An. gambiae* occurred in both types of control huts but was notably higher at Ladji than at Malanville. Both modes of treatment were highly insecticidal at Malanville: ITNs treated with 18 mg/m^2^ lambdacyhalothrin killed 99%, and IRS applied at 30 mg/m^2^ killed 72% of *An. gambiae* that entered the huts. At Ladji, the proportions of *An. gambiae* killed in either the ITN- or IRS-treated hut did not exceed 30% (Table 2).

The proportion of *An. gambiae* collected from the veranda traps in the mornings was greater at Malanville than at Ladji and greater in the huts with untreated nets than in the unsprayed control huts. Relative to the controls, lambdacyalothrin-treated nets and IRS induced little or no exophily of the pyrethroid-resistant *An. gambiae* into the verandas of the Ladji huts, despite high survival rate of mosquitoes in huts. At Malanville, pyrethroid-induced exophily by ITN or IRS hut was not evident and may have been obscured by the high death rates among the mosquitoes.

The personal protection derived from ITN was almost 100% in the susceptible area. Despite the low mortality rate and high rate of blood-feeding observed with ITN in the resistance area, the level of personal protection there was almost 50% because of the deterrent effect of lambdacyhalothin on mosquito entry into huts. The personal protective effect of IRS was low in both areas, and IRS was no barrier to blood-feeding. The overall insecticidal effect of pyrethroid-treated nets and IRS was negligible in the resistance area (<5.4%) but was considerable in the susceptible area (>55.8%).

Table 3 breaks down the mortality data into 2-week blocks. Mortality associated with IRS treatments decreased week by week at both sites but started at a lower rate at the Ladji site because of the expression of resistance. Mortality associated with ITN treatments also showed a downward trend over time at Ladji but not at Malanville, where mosquitoes showed high susceptibility throughout the study.

**Table 3:** Mortality rate of free-flying, naturally entering mosquitoes in huts, first 8 weeks of trial

	Ladji (pyrethroid-resistant *Anopheles gambiae*)						Malanville (pyrethroid-susceptible *An. gambiae*)				
	ITN				IRS		ITN			IRS	
Wk	No.	% Corrected mortality			No.	% Corrected mortality	No.	% Corrected mortality		No.	% Corrected mortality
1–2	41	43.2			15	53.3	67	100	91	100
3–4	83	50.5			42	47.6	93	100	108	88.7
5–6	209	28.7			39	24.2	54	92.6	78	57.8
7–8	53	5.7			21	23.8	53	98.8	118	39.0

*ITN, insecticide-treated net; IRS, indoor residual spraying.

Both ITN and IRS treatments at Ladji showed poor efficacy against *Cx. quinquefasciatus* (this species was not encountered in Malanville). Insecticide-induced deterrence was greater for ITN than for IRS (Table 4). Neither method killed many *Culex* nor stimulated repellency into verandas. The IRS treatment produced an unusually high level of blood-feeding inhibition.

**Table 4:** Experimental hut results of lambdacyalothrin (insecticide)-treated nets (ITNs) and indoor residual spraying (IRS) against *Culex quinquefasciatus*, Ladji (pyrethroid resistance) field station*

Results	Treatments				
	ITNs			IRS	
	Untreated net	Lambdacyhalothrin 18 mg/m^2^		Unsprayed hut	Lambdacyhalothrin 30 mg/m^2^
Total entered	845	598		858	769
Deterred, %	_	29.2		_	10.4
Exiting, % (CI)	29.8 (26.7–32.9)	35.9 (32.1–39.8)		52.7 (49.3–56.0)	54.6 (51.1–58.1)
Blood-fed, % (CI)	62.8 (59.6–56.1)	59.5 (55.6–63.5)		85.1 (82.7–87.5)	42.9 (39.4–46.4)
Blood-feeding inhibition	–	NS		–	49.6
Personal protection, % (no. blood-fed	– (531)	33.1 (355)		– (730)	54.8 (330)
Dead, % (CI)	4.3 (2.9–5.6)	8.5 (6.3–10.8)		3.4 (2.2–4.6)	16.3 (13.7–18.9)
Insecticidal effect, % (no. dead)	– (36)	1.9 (51)		– (29)	11.6 (125)

*CI, 95% confidence interval**.**

### Biochemical Assays and *kdr* Genotyping

*An. gambiae* from Ladji expressed a significantly higher level of oxidase activity than the standard susceptible (Kisumu) and the laboratory *kdr* (Vkper) strains, which had a similar level of oxidase activity. However, the pyrethroid-susceptible strain from Malanville showed a level of oxidase activity that was not significantly different from that of the Ladji strain. This finding would appear to rule out any contribution from oxidases to the pyrethroid resistance observed in *An. gambiae* from Ladji. The level of α-esterase activity in *An. gambiae* from Ladji was significantly higher than that expressed in Malanville or Kisumu strains, whereas the level of β-esterase activity in Ladji, Vkper, and Kisumu strains was similar and clearly played no part in resistance (Table 5). Overall, the mean level of esterase activity at Malanville was significantly lower than that of the susceptible reference strain (p<0.05). Genotyping data (Table 5) showed a high frequency of *kdr* resistance at Ladji (F [*kdr*] = 83%, n = 45) and low frequency at Malanville (F [*kdr*] = 6%, n = 45). The pyrethroid- resistant Vkper was fixed for the *Kdr* gene (F [*kdr*] = 100%, n = 47).

**Table 5:** Efficacy of lambdacyalothrin-treated filter papers* to *Anopheles gambiae* from Ladji, Vkper (fixed for *kdr* allele) and Kisumu (susceptible) strains†

Lambdacyalothrin 0.05% (18 mg/m^2^)–treated filter paper bioassays			
Strains	Slope (SE)	LT_50_ (95% CI)	LT_50_ ratio
Ladji	2.1 (0.2)	10.9 (7.2–14.8)	
Vkper	2.1 (0.2)	14.2 (3.6–25.3)	1.3 (1.0–1.6)
Kisumu		<1	

*In World Health Organization kits.  †As determined by using probit analysis. *kdr*, knockdown; SE, standard error; CI, confidence **interval;** LT_50_ is the exposure time in min to kill 50**%.**

### Adult Bioassays

The summary results of the exposure time mortality bioassays with lambdacyhalothin-treated papers in WHO cylinder kits are shown in Table 6. The slopes and LT_50_s of the probit regression curves were not significantly different for Ladji and Vkper strains. Tests on the Kisumu strain produced 100% mortality after only 1-min exposure. An LT_50_ could not be calculated by using probit analysis, but the resistance factor in the Ladji and Vkper strains was at least 10-fold.

**Table 6:** Molecular and biochemical assays* conducted on samples of *Anopheles gambiae* from Malanville and Ladji compared with laboratory-susceptible (Kisumu) and pyrethroid-resistant *kdr* (Vkper) strains†

Populations or strains	N	Frequency of *kdr* (%)	Oxidase nmol P450 U/mg†	α-esterase μmol/min/mg	β-esterase μmol/min/mg
Kisumu	40	0	0.15 (± 0.020)‡	0.11 (± 0.019)‡§	0.12 (± 0.016)‡
Malanville	45	6	0.25 (± 0.018)§	0.07 (± 0.017)‡	0.04 (± 0.015)§
Ladji	45	83	0.27 (± 0.018)§	0.18 (± 0.017)¶	0.15 (± 0.014)‡¶
Vkper	47	100	0.13 (± 0.017)‡	0.11 (± 0.017)§¶	0.14 (± 0.014)¶

*Mean enzymatic activity ± SE (standard error)**.**  In each column, values not sharing the same superscript are significantly different at the 5% level.

## Discussion

A major loss of efficacy associated with pyrethroid resistance occurred in *An. gambiae* at Ladji, Benin. The reduction in efficacy affected IRS and ITNs equally: only 19% of mosquitoes in the ITN hut and only 22% in the IRS hut were killed after correction for natural mortality. By contrast, 98% of mosquitoes entering the ITN hut and 72% entering the IRS hut located in the susceptible north of Benin were killed by the lambdacyhalothin treatments after correction for natural mortality. These findings are the first clear evidence of pyrethroids’ failing to control an *An. gambiae* population that contains *kdr* resistance at high levels. Whereas the loss of insecticidal effect was calculated to be >95%, a degree of personal protection associated with ITNs and IRS was still evident (45%–50%) relative to the untreated net or unsprayed hut owing to a partial deterrent effect of treatments on entry of mosquitoes rather than to any inhibition of blood-feeding once the insects were inside the huts. Indeed, on entering the huts, most mosquitoes did go on to blood-feed, and the deliberately holed ITN was no barrier to resistant mosquitoes. By contrast, in northern Benin, only 4% of the insecticide-susceptible mosquitoes that entered the hut fed through the holed ITN. The loss of personal protection and loss of mosquito mortality associated with resistance would presumably combine to make ITNs unattractive from the perspective of both the individual user and the malaria control manager. Incision of 80 holes per net is the standard for ITN trials in West Africa (*8*,*9*,*12*), and such nets have given a degree of personal protection in earlier trials. An ITN with no or few holes might be expected to give some protection against resistant mosquitoes from Ladji, but there were insufficient huts available to test this idea.

These experimental hut results from southern Benin stand in contrast to results from an area of Côte d’Ivoire (Yaokoffikro) that had a comparable frequency of *kdr* (78%) to that of Ladji (83%) (*15*) and where lambdacyhalothrin-treated nets and other ITN showed continuing efficacy, with mortality rates of 45%–68% (*8*,*16*–*19*).

We sought evidence that other resistance mechanisms than *kdr* might be contributing to the reduced efficacy of pyrethroids at Ladji. Metabolic resistance due to mixed function oxidases (MFO) has, for example, undermined attempts at malaria control with deltamethrin residual spraying in southern Africa caused by *An. funestus* (*6*), and elevated MFO activity in a strain of *An. gambiae* from Cameroon reduced the efficacy of permethrin-treated netting in laboratory tests (*20*). The combined elevated activity of MFOs, glutathione S–transferase, and esterases resulted in a failure of the S. Mexican IRS program against *An. albimanus* (*21*). Our examination of enzymatic activity in *An. gambiae* showed no evidence that MFO activity is any greater in mosquitoes from Ladji than in mosquitoes from Malanville, nor did esterase activity differ between Ladji and Vkper (*kdr*) strains. Thus, there was no evidence of metabolic resistance enhancing the resistance already caused by *kdr* in mosquitoes from Ladji. Lambdacyhalothin bioassay tests showed no evidence of resistance level differing between Ladji and Vkper strains, and we conclude that metabolic mechanisms made no contribution to the observations in Ladji.

In East Africa a different type of *kdr* based on a leucine-to-serine mutation, which confers resistance to permethrin and DDT (*22*), has been detected in several countries. However, no mosquitoes of this genotype were detected in tests on samples of *An. gambiae* from Ladji (*23*). The complete absence of efficacy of lambdacyalothrin against *Cx. quinquefasciatus* in Ladji merely confirms earlier findings involving other types of pyrethroid in experimental huts in West Africa (*6*,*9*,*16*,*18*).

The contribution of *kdr* to pyrethroid resistance in *An. gambiae* needs to be reappraised. While lambdacyhalothin-treated nets (reported here) and permethrin-treated nets reported earlier (*24*) were less effective in hut trials in the *kdr* area of Benin (Ladji) than in a corresponding area of Côte d’Ivoire (Yaokoffikro), pyrethroid-treated nets were more effective in the susceptible area of Benin (Malanville) than in the corresponding susceptible area of Côte d’Ivoire (M’Be) (*9*) for reasons that are presently unknown. Other differences between the biology of *An. gambiae* from Côte d’Ivoire and Benin exist. Ivorian *An. gambiae* with *kdr* is mainly of the S molecular form, whereas Beninoise *An. gambiae* is of the M form (V. Corbel, unpub. data). M and S forms differ in ecologic distribution and habitat. While mosquitoes of the M form with *kdr* might behave differently from those of the S form with *kdr* when exposed to pyrethroids, this is mere speculation. Moreover, the M form in Malanville showed higher vulnerability to ITN than did the corresponding S form in Côte d’Ivoire, a finding that seems at odds with a behavioral hypothesis.

Our study provides persuasive evidence that pyrethroid resistance in Benin is capable of undermining control measures based on ITN. Nor is there reassurance to be taken from IRS, and any attempt to switch vector control strategy would seem doomed to fail. Whereas the earlier phase 3 malaria control trials of ITN in Côte d’Ivoire showed continuing effectiveness despite *kdr* at high levels (*10*), our phase 2 results from Benin give no grounds for optimism. However, only phase 3 can provide a definitive answer. Further phase 3 trials using pyrethroid-treated nets and IRS need to be undertaken in Benin in an area of pyrethroid resistance. The normal practice with phase 3 is to aim at complete community coverage. Coverage in real life is usually less than total, and the danger with the type of pyrethroid resistance found in Benin is that at lower levels of coverage the important mass protective effect of ITNs (*25*,*26*) may be lost and transmission may continue unabated among those who do not have ITNs. To establish whether this is true, phase 3 trials on resistant mosquito populations should ideally set the coverage level at <100%. If it is considered unacceptable to deny a section of the trial population access to ITNs, an alternative but much less rigorous approach would be to monitor malaria incidence among users and non-users of long-lasting insecticide nets (LLIN) during the proposed scaling up of LLIN coverage in Benin currently being considered.

Pyrethroid resistance in Benin is far from homogeneous, and LLIN should give good protection wherever mosquito populations are susceptible. Use of LLIN should be encouraged but scale-up of treated nets may ultimately select for further resistance. The need to develop alternative insecticides to replace or supplement pyrethroids on nets is urgent and should be put on a par with the seeking of new antimalarial drugs or vaccines that have received far greater attention and resources in recent years.
